# Association of Community Vulnerability and State Gun Laws With Firearm Deaths in Children and Adolescents Aged 10 to 19 Years

**DOI:** 10.1001/jamanetworkopen.2023.14863

**Published:** 2023-05-24

**Authors:** Eustina G. Kwon, Samuel E. Rice-Townsend, Lauren L. Agoubi, Ali Rowhani-Rahbar, Deepika Nehra

**Affiliations:** 1 Division of General and Thoracic Surgery, Seattle Children’s Hospital, University of Washington, Seattle; 2 Division of Trauma, Burn, and Critical Care Surgery, Harborview Medical Center, University of Washington, Seattle; 3 Department of Epidemiology, University of Washington, Seattle

## Abstract

**Importance:**

Firearm-related injuries are the leading cause of death among children and adolescents in the US. For youths aged 10 to 19 years, 64% of firearm-related deaths are due to assault. Understanding the association between the rate of death due to assault-related firearm injury and both community-level vulnerability and state-level gun laws may inform prevention efforts and public health policy.

**Objective:**

To assess the rate of death due to assault-related firearm injury stratified by community-level social vulnerability and state-level gun laws in a national cohort of youths aged 10 to 19 years.

**Design, Setting, and Participants:**

This national cross-sectional study used the Gun Violence Archive to identify all assault-related firearm deaths among youths aged 10 to 19 years occurring in the US between January 1, 2020, and June 30, 2022.

**Exposure:**

Census tract–level social vulnerability (measured by the Centers for Disease Control and Prevention social vulnerability index [SVI]; categorized in quartiles as low [<25th percentile], moderate [25th-50th percentile], high [51st-75th percentile], or very high [>75th percentile]) and state-level gun laws (measured by the Giffords Law Center gun law scorecard rating; categorized as restrictive, moderate, or permissive).

**Main Outcomes and Measures:**

Youth death rate (per 100 000 person-years) due to assault-related firearm injury.

**Results:**

Among 5813 youths aged 10 to 19 years who died of an assault-related firearm injury over the 2.5-year study period, the mean (SD) age was 17.1 (1.9) years, and 4979 (85.7%) were male. The death rate per 100 000 person-years in the low SVI cohort was 1.2 compared with 2.5 in the moderate SVI cohort, 5.2 in the high SVI cohort, and 13.3 in the very high SVI cohort. The mortality rate ratio of the very high SVI cohort compared with the low SVI cohort was 11.43 (95% CI, 10.17-12.88). When further stratifying deaths by the Giffords Law Center state-level gun law scorecard rating, the stepwise increase in death rate (per 100 000 person-years) with increasing SVI persisted, regardless of whether the Census tract was in a state with restrictive gun laws (0.83 in the low SVI cohort vs 10.11 in the very high SVI cohort), moderate gun laws (0.81 in the low SVI cohort vs 13.18 in the very high SVI cohort), or permissive gun laws (1.68 in the low SVI cohort vs 16.03 in the very high SVI cohort). The death rate per 100 000 person-years was higher for each SVI category in states with permissive compared with restrictive gun laws (eg, moderate SVI: 3.37 vs 1.71; high SVI: 6.33 vs 3.78).

**Conclusions and Relevance:**

In this study, socially vulnerable communities in the US experienced a disproportionate number of assault-related firearm deaths among youths. Although stricter gun laws were associated with lower death rates in all communities, these gun laws did not equalize the consequences on a relative scale, and disadvantaged communities remained disproportionately impacted. While legislation is necessary, it may not be sufficient to solve the problem of assault-related firearm deaths among children and adolescents.

## Key Points

**Question:**

Are community-level factors and state-level gun laws associated with rates of firearm-related deaths in children and adolescents?

**Findings:**

In this cross-sectional study including 5813 youths aged 10 to 19 years who died of an assault-related firearm injury, death rates increased in a stepwise fashion with increasing community-level social vulnerability; this trend persisted among all types of state gun laws. States with restrictive gun laws had lower rates of assault-related firearm deaths among youths; however, youths from socially vulnerable communities were disproportionately impacted across the spectrum of state gun laws.

**Meaning:**

These findings suggest that legislation may not be sufficient to solve the problem of assault-related firearm deaths among children and adolescents.

## Teaser

This cross-sectional study assesses the rate of death due to assault-related firearm injury stratified by community-level social vulnerability and state-level gun laws in a national cohort of US youths aged 10 to 19 years.

## Introduction

In the US, firearm-related injuries are now the leading cause of death among children and adolescents aged 1 to 19 years.^1^ Assault-related violence accounts for 64% of firearm-related deaths in youths aged 10 to 19 years.^2^

Social vulnerability indices (SVIs), which measure socioeconomic and population characteristics of discrete geographic regions, are emerging as a useful collective measure of social and societal determinants of health. Previous studies^3,4^ have found that children living in socially vulnerable and disadvantaged communities are at increased risk of violent firearm-related injury. Higher social vulnerability has also been correlated with higher overall injury-related fatality rates.^5^ To our knowledge, the association between community-level social vulnerability and assault-related firearm deaths among youths on a national level has not been previously reported. This lack of research is at least partly because access to detailed data on locations of firearm-related deaths at a national level is limited. The Gun Violence Archive (GVA) maintains an online archive of firearm-related violence incidents detailing the precise locations of firearm-related injuries and deaths, presenting an opportunity to evaluate this association.^6^

In the US, states with more restrictive gun laws have been reported to have lower rates of firearm-related violence^7^; similarly, findings from a systematic review^8^ suggest that in certain nations, the simultaneous implementation of laws targeting multiple firearm restrictions is associated with a reduction in firearm-related deaths. However, it is not understood whether the strength of state-level gun laws has differential consequences for the rate of firearm-related violence in communities with different levels of social vulnerability and disadvantage. We sought to assess the rate of death due to assault-related firearm injury stratified by community-level social vulnerability and state-level gun laws in a national cohort of youths aged 10 to 19 years.

## Methods

### Study Design and Population

This study used publicly available deidentified data and did not require institutional review board approval per guidelines on human participant research from the University of Washington.^9^ The study followed the Strengthening the Reporting of Observational Studies in Epidemiology (STROBE) reporting guideline for cross-sectional studies.

The GVA was used to identify all assault-related firearm deaths among youths aged 10 to 19 years occurring between January 1, 2020, and June 30, 2022. The GVA is a nonpartisan nonprofit organization dedicated to maintaining an online archive of gun violence incidents collected from more than 7500 commercial, governmental, law enforcement, and media sources. It provides near real-time data that capture details surrounding each incident, including date, street location, age, sex, and intention (eg, murder, suicide, domestic violence, or police action).^6^ The GVA records of gun violence deaths have been correlated (*r* = 0.95) with firearm-related deaths reported by the Centers for Disease Control and Prevention,^10^ confirming GVA records to be a reliable source of assault-related firearm deaths. Incidents with no identifiable address and deaths related to suicide were excluded.

### Study Variables

Social vulnerability was determined using the SVI developed by the Centers for Disease Control and Prevention^11^; this SVI provides a comprehensive ranking of the relative vulnerability of every US Census tract, with higher values indicating greater vulnerability. The SVI captures the potential negative consequences for the community due to the aggregate of demographic and social factors in 4 domains: (1) socioeconomic status, (2) household composition and disability, (3) racial and ethnic minority status and language, and (4) housing type and transportation. The Census tract of each incident was determined by converting the incident addresses provided in the GVA to geographic coordinates, which were then matched to Census tract–level geographic identifiers using the Census geocoder.^12^ The geographic identifiers were then linked to the Census tract–level SVI using the 2018 Census tract rankings. Youths were categorized into the following quartiles based on composite SVI: low SVI (<25th percentile), moderate SVI (25th-50th percentile), high SVI (51st-75th percentile), and very high SVI (>75th percentile). Data on youth race and ethnicity categories were not collected because this information is not available through the GVA.

Population estimates of youths aged 10 to 19 years by Census tract in 2020 were obtained from the Surveillance, Epidemiology, and End Results Program (SEER) database^13^ and used for population adjustment. The Giffords Law Center (hereafter, Giffords) 2020 and 2021 gun law scorecard (which categorizes ratings as A, B, C, D, and F) was used to classify Census tracts by state as having restrictive (A and B ratings), moderate (C rating), or permissive (D and F ratings) firearm laws.^14^

### Statistical Analysis

Estimated death rates by SVI category were calculated using the total number of deaths in each SVI category (extracted from the GVA) and per 100 000 person-years from the 2020 SEER population estimates within each SVI category across the 2.5-year study period. Crude mortality rate ratios were calculated, with the low SVI category as the reference group. A similar model was used to estimate the death rates and mortality rate ratios across the SVI categories and Gifford scorecard ratings. All Census tracts were included to estimate person-years regardless of whether the tract had any deaths reported. A Poisson regression model was used to test the interaction between SVI category and Giffords state-level gun law scorecard rating. The linear plots and their slopes were estimated from linear regression analysis of death rates per 100 000 person-years over incident month and year. Changes in death rates over time were assessed using the Mann-Kendall trend test and the Theil-Sen median slope estimator. All analyses were performed using Stata software, version 15/IC (StataCorp LLC). The threshold for statistical significance was 2-tailed *P* = .05.

## Results

We used the GVA to identify 6154 youths aged 10 to 19 years who died due to an assault-related firearm injury between January 2020 and June 2022. After excluding youths who died of suicide (n = 248), incidents without an address (n = 85), and incidents in Census tracts without SVI data (n = 8), 5813 individuals were included in the SVI analysis. For analyses related to Giffords gun law scorecard ratings, an additional 61 deaths due to incidents occurring in an area without a Giffords scorecard rating (ie, Washington, District of Columbia) were excluded, leaving 5752 youths included.

Among 5813 youths, the mean (SD) age was 17.1 (1.9) years; 4979 (85.7%) were male and 834 (14.3%) were female. Using SEER population estimates, the total population of youths aged 10 to 19 years in each SVI quartile was similar (Table 1). The total number of assault-related firearm deaths among youths in the low SVI cohort was 309 compared with 633 in the moderate SVI cohort, 1306 in the high SVI cohort, and 3565 in the very high SVI cohort. The death rate per 100 000 person-years for the low SVI cohort was 1.2 compared with 2.5 for the moderate SVI cohort, 5.2 for the high SVI cohort, and 13.3 for the very high SVI cohort, representing an 11-fold higher death rate in communities with very high social vulnerability compared with communities with low social vulnerability. Compared with the low SVI cohort, the mortality rate ratio was higher in each SVI cohort, with a mortality rate ratio of 2.16 (95% CI, 1.88-2.48) for the moderate SVI cohort, 4.46 (95% CI, 3.94-5.07) for the high SVI cohort, and 11.43 (95% CI, 10.17-12.88) for the very high SVI cohort. Incidents among youths in communities with very high SVI accounted for 25.9% of the total youth population and 61.3% of assault-related firearm deaths (Table 1). Although there was seasonal variation in the monthly death rate per 100 000 person-years during the study period, death rates per 100 000 person-years increased over time in the high SVI communities, with a median slope of 0.060 (95% CI, 0.173-0.581; *P* < .001). Trends in other SVI communities were not statistically significant due to seasonal variation in the number of deaths over the 2.5-year study period; however, a steady increase in the number of deaths over time was seen in all SVI communities with positive slopes in the linear regression model for each SVI category (Figure).

**Table 1.** Frequency and Rate of Death Due to Assault-Related Firearm Injury by Composite Community-Level Social Vulnerability Index

Composite SVI	Frequency	Population aged 10-19 y	Death rate per 100 000 person-years	Mortality rate ratio (95% CI)
Low (<25th percentile)	309	10 639 381	1.2	1 [Reference]
Moderate (25th-50th percentile)	633	10 083 275	2.5	2.16 (1.88-2.48)
High (51st-75th percentile)	1306	10 078 635	5.2	4.46 (3.94-5.07)
Very high (>75th percentile)	3565	10 738 942	13.3	11.43 (10.17-12.88)

Abbreviation: SVI, social vulnerability index.

**Figure.**
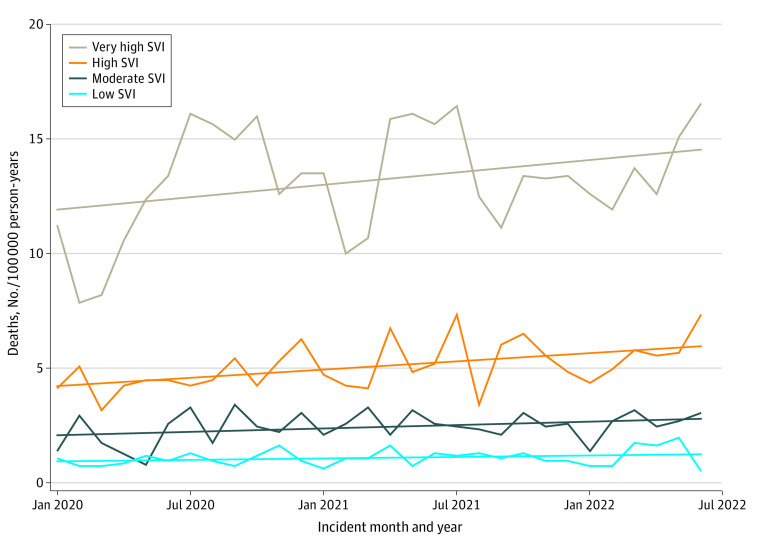
Monthly Assault-Related Firearm Death Rates Among Youths Aged 10 to 19 Years by Composite Community-Level Social Vulnerability Index (SVI) Low SVI indicates lower than 25th percentile; moderate SVI, 25th to 50th percentile; high SVI, 51st to 75th percentile; and very high SVI, higher than 75th percentile.

Stratifying the overall cohort by Giffords gun law scorecard rating, the death rate per 100 000 person-years for Census tracts in states with restrictive gun laws (Giffords A and B ratings) was 4.21 compared with 4.95 for Census tracts in states with moderate gun laws (Giffords C rating) and 7.04 for Census tracts in states with permissive gun laws (Giffords D and F ratings). The mortality rate ratios were 1.17 (95% CI, 1.08-1.27) for Census tracts in states with moderate gun laws and 1.67 (95% CI, 1.57-1.77) for Census tracts in states with permissive gun laws compared with Census tracts in states with restrictive gun laws. Population-adjusted death rates and mortality rate ratios stratified by Giffords state-level gun law scorecard rating and composite SVI quartile are shown in Table 2. There was an increase in death rate (per 100 000 person-years) with increasing SVI regardless of whether the Census tract was in a state with restrictive gun laws (0.83 in the low SVI cohort vs 10.11 in the very high SVI cohort), moderate gun laws (0.81 in the low SVI cohort vs 13.18 in the very high SVI cohort), or permissive gun laws (1.68 in the low SVI cohort vs 16.03 in the very high SVI cohort). The death rate per 100 000 person-years was higher for each SVI category in states with permissive gun laws compared with states with restrictive gun laws (eg, moderate SVI: 3.37 vs 1.71; high SVI: 6.33 vs 3.78) (Table 2). The SVI category and Giffords gun law scorecard rating were found to interact in their association with assault-related firearm deaths among youths (Poisson regression model: *P* = .009).

**Table 2.** Death Rates and Mortality Rate Ratios by Composite Community-Level Social Vulnerability Index and State-Level Giffords Gun Law Scorecard Rating

Composite SVI	Giffords A and B ratings^a^		Giffords C rating^a^		Giffords D and F ratings^a^	
	Death rate per 100 000 person-years	Mortality rate ratio (95% CI)	Death rate per 100 000 person-years	Mortality rate ratio (95% CI)^a^	Death rate per 100 000 person-years	Mortality rate ratio (95% CI)^a^
Low (<25th percentile)	0.83	1 [Reference]	0.81	0.97 (0.66-1.41)	1.68	2.01 (1.55-2.62)
Moderate (25th-50th percentile)	1.71	2.05 (1.58-2.69)	2.04	2.45 (1.84-3.28)	3.37	4.04 (3.20-5.15)
High (51st-75th percentile)	3.78	4.54 (3.59-5.79)	4.82	5.79 (4.52-7.45)	6.33	7.59 (6.09-9.54)
Very high (>75th percentile)	10.11	12.13 (9.79-15.19)	13.18	15.81 (12.67-19.92)	16.03	19.23 (15.57-24.01)

Abbreviation: SVI, social vulnerability index.

^a^ State-level gun law scorecard ratings from the Giffords Law Center, with A and B ratings indicating restrictive gun laws, C indicating moderate gun laws, and D and F ratings indicating permissive gun laws.

## Discussion

This national cross-sectional study found that Census tracts with higher levels of social vulnerability experienced a disproportionate number of assault-related firearm deaths among youths. Communities with very high social vulnerability had an 11-fold higher death rate than communities with low social vulnerability. Although the assault-related firearm death rate among youths was higher overall in states with permissive compared with restrictive gun laws, the increase in death rate with increasing social vulnerability persisted, regardless of restrictive, moderate, or permissive gun laws.

Our findings on the association between community-level social vulnerability and assault-related firearm deaths among youths add to the growing body of literature focusing on the troubling associations between neighborhood disadvantage, lack of opportunity, and violence.^3,4,10,15,16^ Previous studies have described higher numbers of firearm injury-related emergency department visits,^3,4^ increased urban firearm violence,^16^ and increased fatal police shootings^17^ with increased community-level social vulnerability. There is likely a complex array of social phenomena that contribute to these associations. Children and adolescents living in disadvantaged communities are often exposed to lack of economic resources and opportunity, reduced social investment, lack of safe and green spaces, and high levels of community disorganization.^18,19,20,21^ These community-level factors combined with individual- and family-level instability can disrupt normal parent-child relationships and contribute to high-risk behaviors in youths.^22^ The combination of a lack of opportunity and hopelessness may be a common pathway to high-risk behaviors and violence. Our study revealed that these same disadvantaged communities experienced a disproportionate number of assault-related firearm deaths among youths.

To our knowledge, this study is one of the first to describe assault-related firearm deaths among youths by both community-level vulnerability and state-level gun laws. We found the strength of a state’s gun laws to be associated with the rate of assault-related firearm deaths among youths; specifically, stricter gun laws were associated with a lower rate of assault-related firearm deaths. However, these gun laws did not seem to equalize the consequences on a relative scale, as community-level disadvantage was associated with a disproportionate number of assault-related firearm deaths among youths across the spectrum of state gun laws. Our findings suggest that more restrictive firearm laws are unlikely by themselves to reduce the disparities observed in firearm death rates among youths across communities. A previous systematic review^8^ suggested that very few of the existing state-specific firearm laws are associated with reductions in firearm-related mortality; however, this finding must be interpreted with caution because there are limited high-quality studies on the association between firearm legislation and injury, limiting our understanding of the true impact of specific firearm legislation.^23^ Notably, some firearm laws, specifically those focused on the purchase, access, and use of firearms, have been associated with reductions in firearm-related mortality.^8^ Our results suggest that legislation alone, although important, will not address the problem of gun violence in the US and needs to be accompanied by genuine, deep, and long-term investment in historically marginalized communities to reduce inequities.

### Limitations

This study has limitations. First, this is an ecological study, and measures of exposure are based on the mean in the population; thus, our results do not necessarily apply at the individual level. Second, we used the GVA to identify all assault-related firearm deaths among youths. Although the GVA is a rigorously maintained online archive of gun violence–related incidents, there is the potential for both missed events and misclassification,^6^ and detailed demographic data are not available. Third, the GVA records the incident address, and our results describe only the association between the community-level vulnerability of the incident location and youth death related to firearm violence, which may be different from the vulnerability of the community of residence. Fourth, the death rates were calculated using the most up-to-date population estimates available from the SEER database. These SEER database population estimates were for 2020 and were used for the study period extending from January 2020 to June 2022. Changes in the total population of youths aged 10 to 19 years that may have occurred during the study period could not be accounted for. Fifth, this is a national cross-sectional study based on US data; thus, the results are not generalizable to other countries.

## Conclusions

This cross-sectional study of youths who died of assault-related firearm injury found that socially vulnerable communities in the US experience a disproportionate number of assault-related firearm deaths among youths. Although stricter state-level gun laws were associated with decreases in death rates in all communities, the rates in disadvantaged communities remained disproportionately higher, regardless of the strength of the gun laws. Thoughtful and sincere investment in the most disadvantaged communities with the aim of creating opportunity and building community health and safety should be considered an important public health strategy to successfully reduce youth gun violence.

## Notes

Supplement.
Data Sharing Statement
