# Supplementary material for: Association of Community Vulnerability and State Gun Laws With Firearm Deaths in Children and Adolescents Aged 10 to 19 Years
Source: JAMA Netw Open. 2023 May 24;6(5):e2314863. doi: 10.1001/jamanetworkopen.2023.14863 (PMC10209744; doi:10.1001/jamanetworkopen.2023.14863)
Supplement: Supplement. — Data Sharing Statement [file jamanetwopen-e2314863-s001.pdf]

Data Sharing Statement

Kwon. Association of Community Vulnerability and State Gun Laws With Firearm Deaths in Children and Adolescents Aged 10 to 19 Years. *JAMA Netw Open*. Published May 24, 2023. doi:10.1001/jamanetworkopen.2023.14863

Data
Data available: Yes
Data types: Deidentified participant data
How to access data: This data can be obtained upon reasonable request from the corresponding author at: deepikan@uw.edu
When available: With publication

Supporting Documents
Document types: Statistical/analytic code
How to access documents: This information can be obtained from the corresponding author at: deepikan@uw.edu
When available: With publication

Additional Information
Who can access the data: Upon any reasonable request.
Types of analyses: For any purpose.
Mechanisms of data availability: This information can be obtained from the corresponding author at: deepikan@uw.edu
